# Hyper-dimensional computing for enhanced label-free particle analysis in a flow-based optical detection system

**DOI:** 10.1038/s41598-026-44705-z

**Published:** 2026-03-25

**Authors:** Yuanli Yue, Muhammed Gouda, Satoshi Sunada, Peter Bienstman

**Affiliations:** 1 Photonics Research Group, Ghent University-imec, Technologiepark-Zwijnaarde 126, 9052 Ghent, Belgium; 2 Institute of Science and Engineering, Kanazawa University, Kakuma-machi, Kanazawa, 920-1192 Japan

**Keywords:** Engineering, Mathematics and computing, Optics and photonics

## Abstract

Flow-based optical detection is a versatile analytical technique widely used in high-throughput characterization of particles in microfluidic environments. However, conventional implementations often rely on fluorescent labeling or bulky imaging hardware, which can be time-consuming, costly, and potentially harmful to cell viability. To address these challenges, label-free imaging combined with brain-inspired computational approaches have emerged as promising alternatives. In this study, we present a label-free particle analysis framework that integrates Hyper-Dimensional Computing (HDC) with an event-based imaging system for fast and accurate classification of microparticles. A proof-of-concept experiment is performed using an event-based camera to capture optical interference patterns generated by microparticles of four different sizes through a polymethyl methacrylate (PMMA) microfluidic channel. HDC is then employed in the post-processing stage to classify these event-derived patterns efficiently, with a low computational overhead. To further enhance optical diversity and improve classification accuracy, a ground-glass diffuser is introduced into the optical path. Comparative experiments across multiple ground-glass diffuser configurations show that the classification accuracy can reach up to 98.67% under the best diffuser condition. These findings demonstrate the feasibility of combining HDC and event-driven photonic detection for compact, label-free classification of synthetic microparticles under controlled experimental conditions. While the current study is limited to polystyrene beads with well-defined size differences, the proposed framework provides a basis for future investigations toward more complex biological or industrial particulate systems.

## Introduction

Flow-based optical detection systems provide a powerful platform for rapid, high-throughput analysis of individual microparticles based on their size, shape, and internal complexity^1^. By enabling single-particle measurements in microfluidic environments, these systems support a wide range of applications, from environmental monitoring to medical detection in cell sorting^2,3^. In medical research, such systems are particularly valuable for detecting cellular heterogeneity, identifying rare populations, and monitoring dynamic biological processes in real time. Consequently, flow-based optical detection techniques have become essential tools across fields including biomedical research, clinical diagnostics, immunology, and oncology^3^.

Optical detection techniques can be broadly classified into label-based and label-free methods^4,5^. Traditional label-based approaches typically rely on fluorescence, requiring the tagging of biological or particulate components–such as proteins or surface markers–with fluorescent dyes. As the labeled samples pass through a laser beam, the emitted fluorescence signals are detected and analyzed, enabling detailed multi-parametric classification^6^. Although this method provides high sensitivity and accuracy, it also introduces some drawbacks: fluorescent labeling can stress cells, alter sample properties, and is time-intensive and costly. As a result, label-free-based optical detection has emerged as a promising approach for fast, cost-effective, and non-invasive particle analysis^7^.

Label-free detection methods eliminate the need for analyte modification, and instead exploit the intrinsic optical properties of the target, such as light scattering, diffraction, or interferometric phase contrast. These methods enable characterization of particle features–such as size, shape, and refractive index–directly from optical signals without biochemical labeling. Among various label-free cytometry techniques, pattern acquisition is typically achieved through optical scattering, interferometry, diffraction imaging, quantitative phase imaging (QPI), and optical coherence tomography (OCT)^5,8,9^. These techniques can be combined with microfluidics to improve compactness, reduced reagent consumption, and improved compatibility with automated analytical platforms^10^. By leveraging intrinsic particle or cellular properties– such as refractive index, morphology, and internal structure–offering non-invasive and high-resolution measurement capabilities without the need for fluorescent labels.

Despite these advantages, label-free optical detection systems face significant challenges, particularly in handling and processing the large volumes produced at high acquisition rates. Extracting discriminative features from dynamic optical patterns in real time remains a key bottleneck. Conventional frame-based imaging systems often suffer from high latency, redundancy, and computational overhead, limiting their ability to process high-speed flow data efficiently^11^. Existing scattering- and diffraction-based methods can capture morphological signatures of particles but generally rely on conventional frame-based cameras, which are limited by motion blur and data redundancy at high flow rates. Meanwhile, QPI and OCT provide rich phase information but require complex optical setups, long acquisition times, and computationally intensive reconstruction processes. Therefore, efficient feature extraction and classification remain critical bottlenecks for deploying responsive, scalable solutions in clinical and research settings.

To address this, brain-inspired computational paradigms, such as HDC, provide a promising solution. HDC encodes input features into high-dimensional binary vectors–so-called hypervectors–allowing for fast, noise-robust, and energy-efficient classification^12,13^. Compared with conventional neural networks, HDC performs lightweight operations while maintaining competitive accuracy, making it highly suitable for embedded or low-power optical sensing systems^14^.

In this work, we present a label-free particle analysis framework that integrates HDC with an event-based imaging system. The event-based camera records optical patterns produced by microparticles flowing through a PMMA microfluidic channel under laser illumination. Unlike conventional CMOS sensors, event-based imagers operate asynchronously and register only changes in brightness, producing sparse, high–temporal-resolution data that emphasize dynamic scattering features while minimizing redundancy. The captured event patterns are subsequently processed by HDC to perform efficient classification. In this study, HDC encodes the acquired interference patterns into a high-dimensional binary space, where class-representative hypervectors are trained to distinguish between four microparticle size classes. Both positive and negative polarity events recorded by the event-based camera are analyzed to evaluate their impact on classification performance. To reduce measurement bias and enhance accuracy, multiple measurements are conducted and analyzed in an interleaved manner.

To further enhance optical diversity and improve classification accuracy, we introduce a ground-glass diffuser between the microchannel and the detector, which enriches the optical interference and scattering characteristics of the captured patterns. Comparative experiments with diffusers of varying grit values reveal that coarser scattering surfaces yield broader and more distinctive optical signatures, improving classification performance.

This study demonstrates a proof-of-concept implementation of event-driven, HDC-based label-free analysis allowing to classify beads of different sizes. The approach combines optical sensing and efficient computation to enable accurate, low-cost, and scalable detection, providing a methodological foundation for future studies investigating more complex biological or industrial particulate systems.

## Results

### Measurement bias

In general, supervised machine learning algorithms aim to perform specific tasks–such as classification or prediction–by learning from a given set of labeled training samples. However, one common challenge in such algorithms is measurement bias, which arises when noise in the data is correlated with the training labels. This often occurs when the datasets for different classes are collected under different measurement conditions or experimental setups.

Measurement bias can lead the model to mistakenly interpret differences in measurement conditions as distinguishing features of the classes. As a result, models may achieve deceptively high classification accuracy when both training and test samples are affected by the same bias, masking the true generalization performance.

To address this issue, Lugnan^15,16^ proposed an intertwined measurement scheme that successfully reduced measurement bias in flow cytometry. He highlighted several potential sources of drift in measurement parameters, such as fluctuations in the light source intensity, optical beam distortions (e.g., from thermal expansion), and changes in the refractive index of optical components (e.g., due to water absorption in microfluidic channel walls).

Inspired by this approach, our experiment adopts this intertwined measurement scheme to mitigate measurement bias. Indeed, for each class, we conduct data acquisition under a variety of different measurement conditions, in practice corresponding to different measurement sessions at different moments in time. We evaluate the system performance using two different approaches, namely single measurement and intertwined measurements. In the single-measurement approach, data from each class is collected only once (first measuring class A, then B, C and D), and could potentially suffer from measurement bias due to different experimental conditions, leading to an overestimation of the performance. For the intertwined approach, we collect a much larger dataset by performing measurements first of class A, then B, C and D, and then restarting to measure A, B, C and D several more times, such that each class has measurement data under different experimental conditions. This should lead to a much fairer estimation of the generalization performance of the flow cytometry system.

In our experiment, each particle class was measured six times, resulting in six separate datasets per class (e.g., A1, A2, A3, A4, A5, A6). For single measurement classification, both the training and testing datasets are taken from the same measurement instance. In contrast, for intertwined measurement classification, the training and testing datasets come from different measurement instances.

### Classification without a scattering medium

**Fig. 1:**
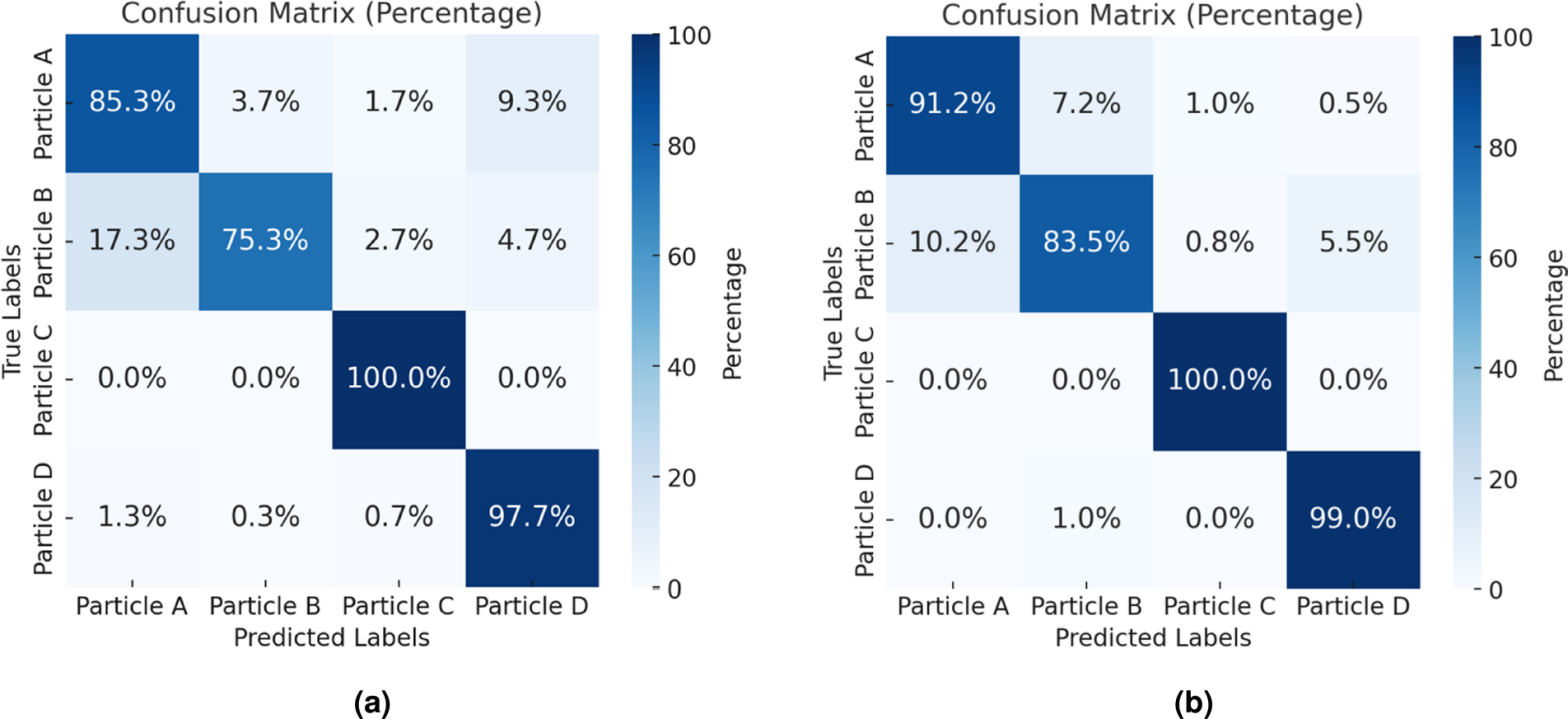
Confusion matrix for single-session measurements: (**a**) classification without considering polarity. (**b**) classification result with polarity.

We begin with single-session measurement classification, where each class is measured only once, potentially being subjected to drift as we measure different classes. Both the training and testing datasets are collected from the same dataset. In this work, the dimension of HDC is set to D = 10,000. In practice, a total of 24,000 event samples (6,000 per particle type: A–D) were collected for training, and 2,400 samples (600 per type) were used for testing. Each event corresponds to a single microparticle passing through the illumination region under identical flow conditions. The data were balanced across classes to ensure fair comparison. The resulting confusion matrix is shown in Fig.  1. We compared two approaches: with and without polarity consideration in single-session measurements. Including polarity improves performance, as it provides additional discriminative information. In our implementation, polarity information is incorporated during the pre-processing stage. Each event frame is decomposed into two binary maps representing positive and negative events, corresponding respectively to local intensity increases and decreases detected by the event camera. These two maps are then concatenated to form a single extended binary vector, effectively doubling the feature dimension. The resulting hyper-vector thus contains spatial and polarity-specific information, allowing the HDC model to capture finer variations in scattering patterns. When polarity is ignored, the two maps are merged into a single binary representation, which reduces discriminative detail and leads to a lower classification accuracy. Without polarity, the classification accuracy is approximately 89.58%, whereas incorporating polarity increases the accuracy to 93.06%.

To evaluate the effect of bias, we compared the results from single-session and multi-session (intertwined) measurements. The single-session model achieved an accuracy of up to 95.75%, while the intertwined model reached 93.42%. Although the accuracy is slightly lower, the intertwined model helps mitigate measurement bias, offering a more robust generalization and a fairer assessment of the performance. Among the four particle types, particle B (12 $\upmu$m) exhibited slightly lower classification accuracy. This can be attributed to the fixed pixel size of the event-based camera, which may cause the 12 $\upmu$m particle patterns to misalign with the camera’s resolution. While smaller particles (e.g., 9 $\upmu$m) generate clearer, more distinguishable patterns, and larger particles (e.g., 16 $\upmu$m and 20 $\upmu$m) produce more defined features, the interference patterns of intermediate-sized particles like 12 $\upmu$m are harder to resolve, leading to reduced accuracy.

### Classification with the scattering medium

Using the same experimental setup, we evaluate and compare the classification performance across diffusers with different grit levels. For each diffuser condition (120, 600, and 1500 grit), five independent measurement sessions were conducted, and the reported accuracy corresponds to the mean value across these five experiments. Each accuracy value in Table 1 represents the averaged classification performance across the four particle types (A–D). To minimize potential systematic bias arising from temporal drift or environmental variations, the order of diffuser testing was randomized across experimental sessions. The results are presented in Table 1.

**Table 1:** Comparison of the accuracy with different grit levels. Intertwined measurements were used.

Condition	Accuracy
No diffraction grating	93.42%
Ground Glass (120 Grit)	98.67%
Ground Glass (600 Grit)	96.67%
Ground Glass (1500 Grit)	90.25%

The best performance was obtained using a grit of 120. From Fig.  7, we know that this grit value results in a wider diffraction pattern with more accuracy, which explains the improved performance. It should be noted that the current study uses microparticles with relatively large size differences (9–20 $\upmu$m). While commercial flow cytometers and other label-free cytometry techniques can effectively differentiate particles with size differences as small as 3 $\upmu$m, our method offers key advantages, particularly for label-free applications. Our approach uses an event-based camera that captures only relevant frames, reducing the computational load compared to traditional systems. By recording only changes in the scene, it enables faster, real-time analysis and higher throughput.

### Impact of feature dimensions

**Fig. 2:**
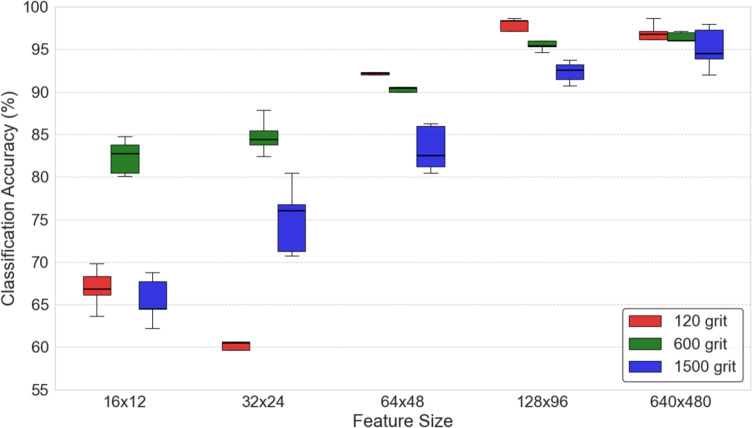
Classification accuracy of different grit sizes across various feature sizes D.

In our experiment, interference patterns were initially captured at a resolution of 640 $\times$ 480 pixels (i.e., 640 $\times$ 480 features). Prior to post-processing, we also investigated uniform down-sampling, enabling a comparison of classification performance across varying feature dimensions D. The impact of different feature dimensions on classification performance is illustrated in Fig.  2. For each feature dimension, classification accuracy is evaluated separately for each diffuser condition (120, 600, and 1500 grit), and the curves shown in Fig.  2 correspond to these individual grit settings rather than averaged values across diffusers. Increasing feature dimensions generally improves classification performance; however, the gain diminishes at higher resolutions. For instance, under the 1500-grit condition, increasing the feature size from 128$\times$96 to 640$\times$480 pixels improves the accuracy only marginally (from 90.25% to approximately 93%), while substantially increasing computational cost. This indicates that moderate feature resolutions can already achieve competitive performance without unnecessary computational overhead.

#### Comparison with spiking neural networks

Previous research using Spiking Neural Networks (SNNs) has achieved classification accuracies of up to 99% in various microparticle classification tasks^17^. While SNNs deliver excellent accuracy, they require significant computational resources. Their training process is complex, computationally intensive, and time-consuming, especially without hardware acceleration. Thus, while the high accuracy of SNNs demonstrates their strong modeling capability, it comes at the cost of increased computational complexity. SNN training requires iterative surrogate-gradient-based optimization with backpropagation through time over multiple epochs, involving temporal unrolling of spiking activity and repeated weight updates.

In contrast, HDC training consists of a single-pass encoding and prototype bundling procedure without gradient-based optimization. The training process relies primarily on binary hypervector operations and accumulation, leading to linear computational scaling with respect to the number of samples and feature dimensionality. While direct wall-clock training time depends on specific hardware implementation, the absence of epoch-based gradient optimization in HDC inherently results in substantially lower training overhead compared to surrogate-gradient-based SNN training.

Furthermore, inference in SNNs may involve hundreds of thousands to millions of synaptic operations per sample depending on network depth, whereas HDC inference reduces to Hamming distance computation in high-dimensional binary space. Therefore, although the achieved accuracy (up to 98%) is slightly lower than that of SNNs, HDC provides a favorable trade-off between accuracy, computational complexity, and hardware simplicity for real-time flow cytometry applications.

## Discussion

In this study, we experimentally demonstrate a high-throughput flow cytometry system based on HDC and an event-based camera. Our results show that HDC can accurately classify particles at modest computational cost. The introduction of a scattering medium–specifically, ground glass–further improves performance. We investigated three types of ground glass diffusers with different grit levels (120, 600, and 1500) and found that coarser grit (lower grit value) provides better classification results due to broader and more uniform light scattering.

The best classification accuracy achieved in our experiments was 98.67%. Overall, the proposed HDC-based, event-driven flow cytometry system demonstrates strong potential for accurate and efficient microparticle classification. This approach also provides valuable insights for developing label-free flow cytometry techniques, with promising applications in early diagnosis and biomedical research.

In this study, we focused on a proof-of-concept validation using microparticles rather than biological cells. The objective was to evaluate the feasibility of combining event-based sensing with HDC for label-free pattern classification in a controlled environment. Using standard microspheres allows accurate benchmarking of the algorithm and minimizes biological variability. Although the present work is restricted to synthetic microspheres with controlled size variations, the results demonstrate the robustness of the sensing and computational pipeline under well-defined conditions. Extension to biological cells will require further validation, as additional factors such as morphological variability, refractive index heterogeneity, mechanical deformation, and intra-class diversity may significantly influence the optical signatures and classification performance.

In addition, the current experiments were conducted under controlled laboratory illumination. Event-based cameras are inherently sensitive to background light fluctuations and sensor noise, which may generate spurious events under non-ideal environmental conditions. While such effects can be mitigated through stable laser illumination, optical shielding, and preprocessing strategies such as polarity balancing and threshold tuning, real-world or portable deployments may require additional hardware-level stabilization or adaptive threshold control mechanisms to ensure consistent performance.

Furthermore, the intertwined measurement scheme adopted in this work effectively reduces measurement bias and provides a more realistic estimation of generalization performance. However, this strategy increases total data acquisition time compared to single-session measurements. In high-throughput industrial or clinical scenarios, a trade-off may arise between minimizing systematic bias and maximizing throughput. In practice, once system calibration and environmental stability are established, partial or periodic interleaving strategies may provide a balanced compromise between robustness and operational efficiency.

In the present study, all particles were composed of polystyrene, ensuring that classification differences were primarily driven by particle size rather than material properties. In practical applications, heterogeneous particles with varying refractive indices may generate variations in scattering intensity and interference contrast. Since the proposed framework relies on pattern-based encoding rather than explicit physical parameter estimation, it is expected that the HDC classifier can learn material-dependent features when trained on sufficiently diverse datasets. Exploring classification performance on particles with varying refractive indices and heterogeneous material compositions constitutes a possible direction for future research.

Beyond classification, the proposed framework may also be conceptually integrated with optical sorting techniques to enable closed-loop, real-time particle separation. For instance, recent structured-beam-based approaches have demonstrated refractive-index-dependent trapping and propulsion for label-free microparticle sorting using Airy beams^18^. In a hybrid architecture, the event-based camera could first capture the dynamic scattering signature of a flowing particle and perform rapid classification via HDC. The classification output could then trigger an optical actuation module–such as a spatial light modulator (SLM)-controlled structured beam or optical tweezers–to selectively deflect, trap, or redirect the particle within the microfluidic channel. Given the sub-millisecond latency of the proposed detection pipeline, such integration is conceptually compatible with flow speeds typical of optical manipulation platforms. This suggests the possibility of developing a fully optical, detection-and-sorting system in future implementations.

## Methods

**Fig. 3:**
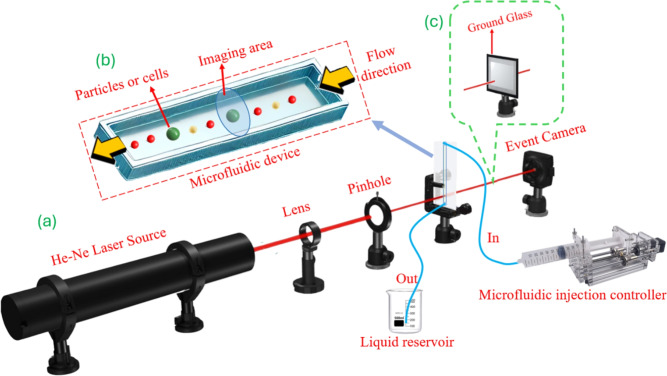
Schematic of the optical detection system utilizing an event-based camera for image acquisition.

The proposed experimental setup is illustrated in Fig. 3a. A He–Ne laser with a central wavelength of 632.8 nm serves as the coherent light source. A lens is placed after the laser source to focus the beam onto a 25 $\mu$m diameter pinhole. Together, the lens and pinhole form a spatial filtering stage that improves the spatial quality and uniformity of the illumination entering the microfluidic channel, thereby enhancing the stability and contrast of the generated diffraction patterns. Positioned immediately downstream, a PMMA microfluidic channel—mounted vertically in the setup—features a square cross-section of 200 $\mu$m $\times$ 200 $\mu$m and a total length of 58.5 mm. PMMA was selected for its excellent optical transparency, ease of fabrication, biocompatibility, and cost-effectiveness, making it well-suited for scalable microfluidic detection systems. The experiment’s silicone tube and the microfluidic channel are purchased from the microfluidic ChipShop GmbH.

Microparticles were introduced into the channel using a high-precision automatic syringe pump, driven by a stepper motor, to maintain a stable and controlled flow from the upper to the lower port. A collection reservoir at the outlet collects the discharged fluid. The imaging region, shown in Fig. 3b, was aligned with the pinhole and defined as a circular area with a diameter of 25 $\mu$m. In this experiment, the particles are pumped using 20 mL manual syringe pumps. During the experiment, we used five different syringes, for particle A, particle B, particle C and particle D, with the fifth one for flushing. The concentration of the microparticle solution was set to 2%. This concentration was selected to ensure an optimal number of particles within the detection area, maintaining a balance between particle density and avoiding overlap that could affect the accuracy of pattern capture. The flow rate was set to 3 mL/min within the 200 $\mu$m $\times$ 200 $\mu$m channel. This moderate flow ensures stable single-particle passage through the detection region while providing sufficient temporal resolution for event-based imaging without introducing motion blur. Flushing is done with deionized water (DI) to avoid introducing impurities. In this work, we also propose another variation with an additional scattering medium in order to improve the classification performance. As shown in Fig. 3c, various ground-glass diffusers with different grit levels were placed between the microfluidic channel and the event-based camera to modulate the optical patterns generated by the flowing particles.

In this study, four types of polystyrene (PS) microparticles with distinct diameters were used: A (9  $\mu$m), B (12  $\mu$m), C (16  $\mu$m), and D (20  $\mu$m). The particles were purchased from PolyAn GmbH (Germany). As each particle flows through the laser beam within the channel, it produces an interference pattern that is captured by an event-based camera (Prophesee EVK1 Gen31) with a resolution of 640 $\times$ 480 pixels. Simultaneously, streaming data from the flow is collected for subsequent analysis. The Prophesee EVK1 Gen31 event-based camera has a sensor size of 1/3 inch (approximately 6.76 mm $\times$ 5.07 mm) corresponding to the resolution of 640 $\times$ 480 pixels. Each pixel corresponds to a physical size of approximately 10.56 $\mu$m. This configuration ensures accurate spatial calibration for the images, allowing for precise capture of dynamic optical patterns generated by the microparticles flowing through the microfluidic channel. All images presented in this paper are captured in their entirety, with the scale bars calibrated according to the physical dimensions of the sensor and pixel size.

This event-based camera, also known as a dynamic vision sensor (DVS), is capable of capturing fast-moving objects and dynamic scenes with sub-millisecond temporal resolution and minimal motion blur. Compared with conventional CMOS or CCD cameras, the event-based sensor offers several advantages for flow-based optical detection. Unlike frame-based cameras that acquire consecutive images at fixed intervals, the DVS detects only changes in the scene. Each pixel operates independently and asynchronously, responding to local brightness variations. This asynchronous sensing mechanism enables sparse, low-redundancy data acquisition that focuses exclusively on relevant motion events. By recording only intensity changes rather than full image frames, the system achieves extremely low latency and significantly reduces data volume, both essential for real-time, high-throughput particle analysis. The resulting events are streamed and stored for frame reconstruction, post-processing, and classification.

However, event-based sensors also present challenges: they are sensitive to background illumination and may generate spurious events under non-uniform lighting conditions. These effects can be mitigated through proper threshold tuning and polarity balancing during preprocessing, but they must be considered when extending the system to more complex biological or scattering environments.

In this study, HDC is employed to analyze and classify the captured interference patterns. HDC efficiently encodes event-derived data into high-dimensional representations, offering noise-robust and energy-efficient processing. The following section introduces the principles of HDC and describes how it is integrated into the experimental workflow to enable accurate classification of the captured patterns.

### Hyper-dimensional computing

HDC is an emerging computational paradigm based on high-dimensional vector representations and algebraic operations. It leverages properties such as robustness, efficiency, and parallelism, making it suitable for tasks involving pattern recognition, classification, and data encoding. HDC has demonstrated promising results in one-dimensional data processing tasks, offering lower power consumption and latency compared to conventional deep neural networks (DNNs)^19^. The HDC framework typically consists of three key stages: encoding, training, and comparison. The primary goal of the encoding stage is to map input data into a high-dimensional space, where feature extraction and pattern recognition become more efficient and robust^13^. During this process, each input is nonlinearly transformed (see section “Classification without a scattering medium” for details) into a binary high-dimensional vector (Hyper-Vector, or HV), which encodes the essential features of the input data. During the training phase, to represent a class, multiple HVs corresponding to different instances of the same class are generated and aggregated. This aggregation is performed through a process known as bundling, which combines individual HVs into a single prototype vector that incorporates features from all constituent HVs. Let $P_l$ denote the prototype HV representing class *l*. Let $\{V_k^l\}_{k=1}^{N_s}$ be the set of HVs corresponding to class *l*, where *k* is the index of the sample and $N_s$ is the number of samples for that class. The bundling operation that generates the prototype HV $P_l$ can be formally expressed as:

1 $$\begin{aligned} P_l = \left( V_1^l + V_2^l + \cdots + V_{N_s}^l \right) \end{aligned}$$

**Fig. 4:**
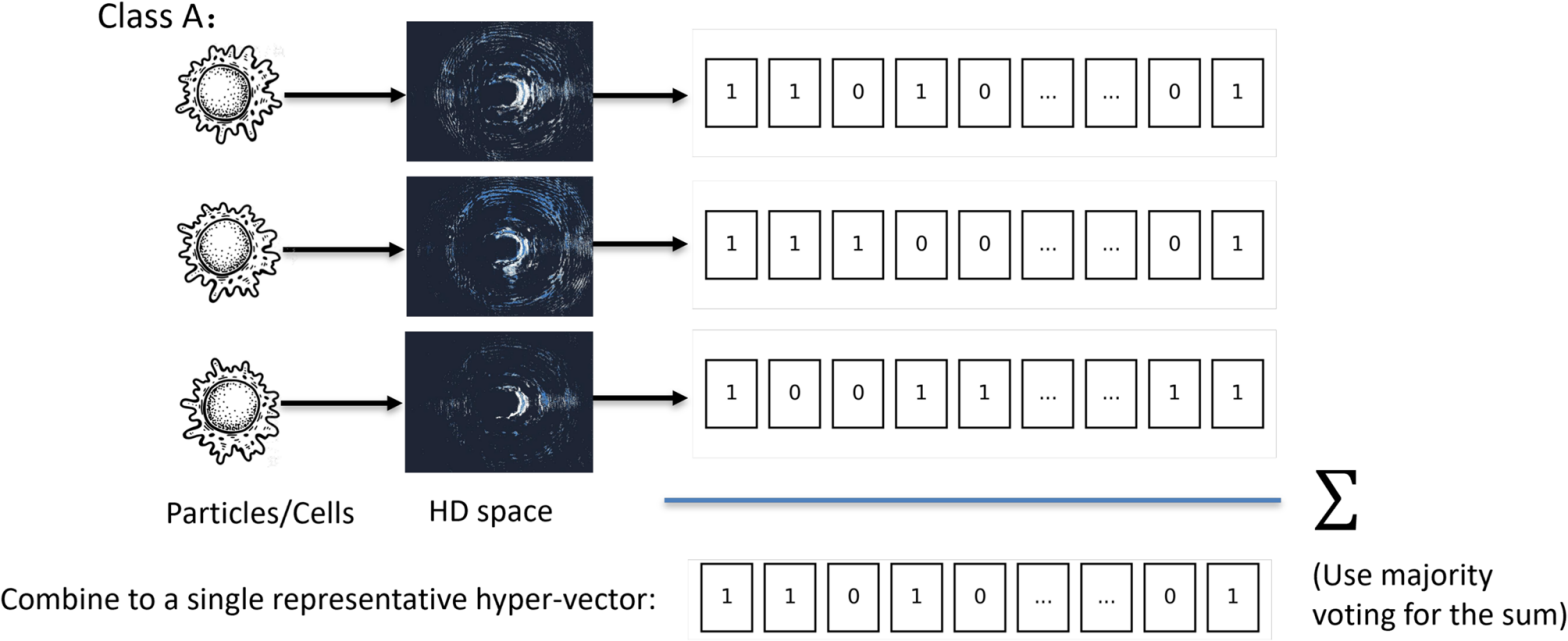
The training pipeline of the HDC.

In our case, for ‘+’, we use bitwise majority voting to generate the prototype hyper-vector, i.e. for each element of the prototype vector, we select the binary value that occurs the most in the training set at that position (see Fig.  4).

During the testing phase, the same encoding process is applied to the input features to generate a HV representing the test sample, referred to as the query vector, Vq. The classification is performed by comparing Vq with all stored prototype HVs using a similarity metric. Two common similarity measures used in HDC are cosine similarity^12^ and Hamming distance^19^. Cosine similarity is appropriate for real-valued HVs, while Hamming distance is more suitable for binary HVs. Since our implementation uses binary HVs, we adopt Hamming distance for classification. The Hamming distance between two binary vectors is defined as the number of positions at which the corresponding vectors differ. Formally, it can be computed as:

2 $$\begin{aligned} \textrm{Ham}(A, B) = \sum _{j=1}^{D} A_j \oplus B_j , \end{aligned}$$

Here, $\oplus$ denotes the bitwise XOR operation, which yields 1 if and only if the corresponding bits $A_j$ and $B_j$ differ, and 0 otherwise. The Hamming distance, $\textrm{Ham}(A, B)$, is equal to 0 only when $A = B$, indicating perfect similarity. In contrast, when *A* and *B* are uncorrelated, the Hamming distance approaches on average 0.5*D*, where *D* is the dimensionality of the HVs^14^.

During classification, an unknown sample is assigned to the class $l^*$ whose prototype vector $P_l$ has the minimum Hamming distance from the query vector $V_q$. This can be expressed as:

3 $$\begin{aligned} l^*= \arg \min _{l} \, \textrm{Ham}(P_l, V_q). \end{aligned}$$

In the following section, we describe how the captured interference patterns are used to generate the hyper-vectors.

### Encoding the hyper-vectors

The encoding process, which converts input data into hypervectors (HVs), is a core component of Hyperdimensional Computing. Traditional methods typically employ arithmetic operations for encoding^13^. For instance, record-based encoding uses two sets of HVs: one for feature positions and another for feature values. To encode a feature vector with m elements, m randomly generated HVs represent the positions, while feature values are discretized into n levels, each mapped to a corresponding HV. These level HVs are constructed to preserve correlations between adjacent levels. Further details on conventional encoding techniques are available in^20^.

However, such arithmetic-based approaches can be computationally intensive. Our proposed method addresses this by replacing arithmetic encoding with an optical scattering process to generate HVs on the fly, where each element of the binary HV corresponds to the presence or absence of events in a certain region of the image. This reduces computational overhead.

**Fig. 5:**
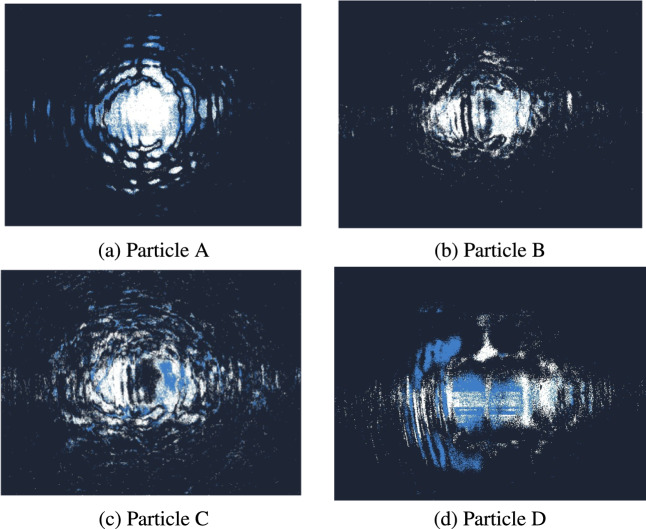
Captured frames of particles with different diameters, including polarity information.

Since we opt for using binary vectors, the post-processing stage must still consider the polarity of event-based cameras. Indeed, these cameras produce both positive (increasing intensity) and negative (decreasing intensity) events, visualized as white and blue pixels, respectively. This information is crucial for capturing the motion patterns of microparticles, as it enhances the dynamic range of event-based data and reveals the motion patterns of microparticles. Fig.  5 illustrates the different particle frames that includes polarity information.

**Fig. 6:**
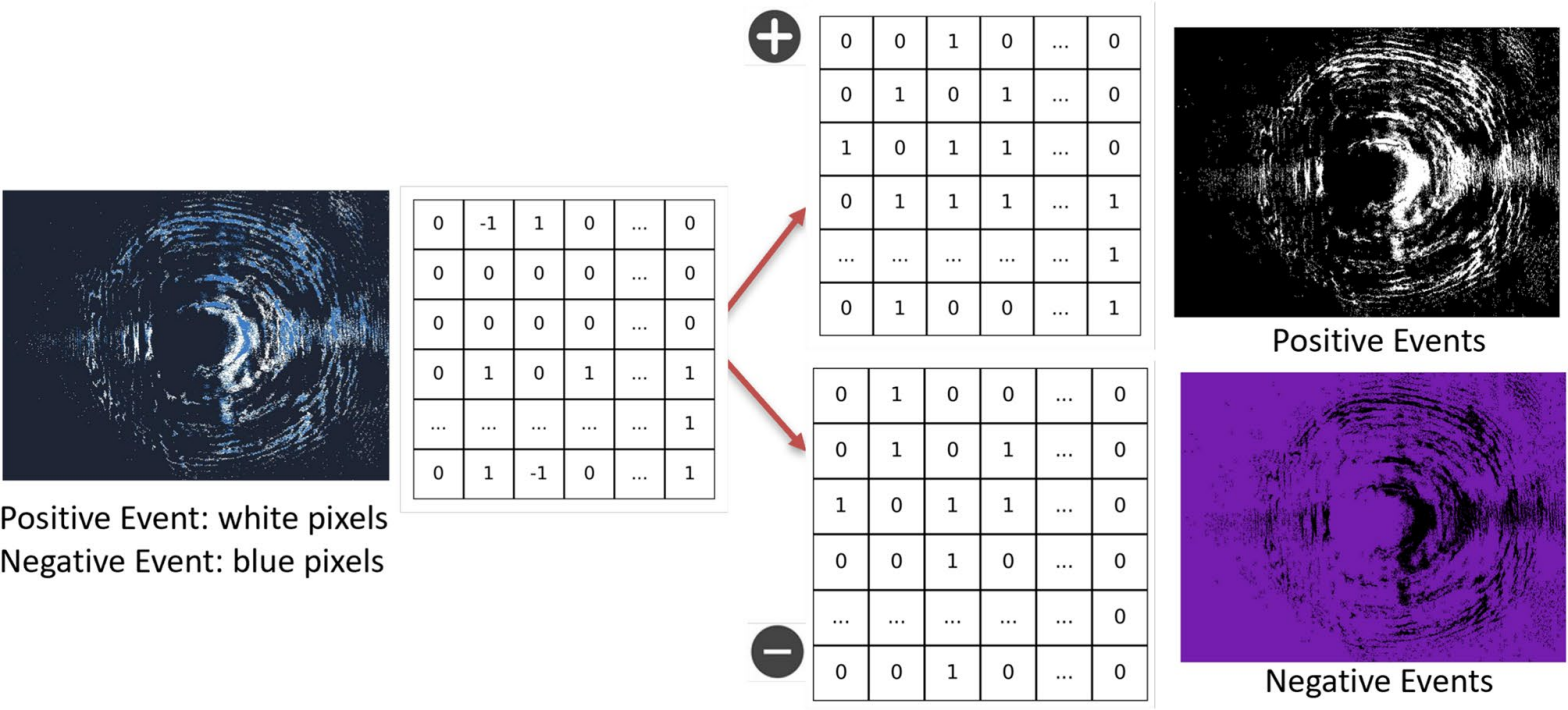
Visualization of the captured frame containing polarity data, along with the extracted positive and negative event components. The left panel shows the reconstructed frame directly from the event-based sensor output. Bright (white) pixels denote positive events (intensity increase), and blue pixels denote negative events (intensity decrease). The right panels separately display the extracted positive and negative components used for HDC encoding.

In the pre-processing stage, both positive and negative events are extracted from the frames, binarized, and concatenated into a single binary vector of twice the original size for HDC processing. The event extraction workflow is illustrated in Fig.  6.

### Scattering medium

**Fig. 7:**
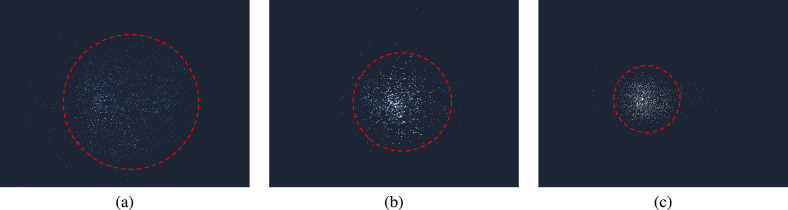
Scattering patterns with different ground glasses ((**a**) 120 Grit; (**b**) 600 Grit; (**c**) 1500 Grit).

In our experiment, a scattering medium is introduced to enhance the dynamic response of the collected interference patterns, as shown in Fig.  3c. Specifically, a ground glass diffuser is placed after the microfluidic channel to scatter the interference patterns. All ground glass diffusers used in our setup were polished diffusers sourced from Thorlabs. Compared to sand-blasted variants, polished diffusers offer greater surface uniformity. We used three types of ground glass with different grit values: 120, 600, and 1500. The selected grit levels provide a spectrum from fine to coarse scattering. A finer grit (e.g., 1500) yields a narrower diffusion pattern, while a coarser grit (e.g., 120) generates a wider one, as illustrated in Fig.  7, where the scattering regions are highlighted with red dashed circles, The impact of different grit values on classification performance will be explored in the following section.

## Data Availability

The datasets used and analysed during the current study available from the corresponding author on reasonable request.
